# Continuous Glucose Monitoring in Older People With Diabetes Mellitus and Cognitive Impairment: A Brief Review

**DOI:** 10.1177/19322968251384992

**Published:** 2025-10-20

**Authors:** Busra Donat Ergin, Kieran Gadsby-Davis, Katharina Mattishent, Ketan Dhatariya, Anne-Marie Minihane, Michael Hornberger

**Affiliations:** 1 Norwich Medical School, University of East Anglia, Norwich, UK; 2 Norfolk and Norwich University Hospital, Norwich, UK; 3 Department of Clinical and Experimental Sciences, Faculty of Medicine, University of Southampton, Southampton, UK

**Keywords:** continuous glucose monitoring, hospitalization, diabetes mellitus, dementia, inpatients

## Abstract

**Background:**

Diabetes mellitus and dementia are common chronic diseases affecting older people in the community and in hospitals. Even though both diseases have been independently well-characterized, comorbid diabetes and dementia/cognitive impairment are much less understood. In particular, cognitive impairment can make glucose monitoring much more challenging and can more readily lead to diabetes-related emergencies such as hypoglycemia, hyperosmolar hyperglycemic state, or diabetic ketoacidosis. Based on this, improving diabetes management in the community and in the hospital settings via glucose monitoring is essential in older people with T2DM and particularly those with comorbid diabetes and dementia.

**Aim:**

The use of continuous glucose monitoring (CGM) holds promise for greater glycemic management in older patients with diabetes and those at high risk for dementia. In this brief review, we will review the few existing studies for CGM use in the community and the hospital in this population, as well as the link between hospital admissions.

**Results:**

Existing studies show high feasibility and good adherence with using CGM among older people. In addition, diabetes technologies can improve risk factors associated with hospitalization, leading to decreased hospitalization rates. We illustrate how the current studies highlight the need for studies in the hospital in this frail population, who potentially will benefit most from CGM systems.

**Conclusion:**

Although existing feasibility studies show high promise in this frail population, more data are needed on CGM for older people living with diabetes and memory problems in the hospital setting.

## Introduction

Diabetes is one of the most common long-term conditions, with approximately 4.6 million people in the United Kingdom having a diagnosis of diabetes mellitus (DM) and an estimated 1.3 million people remaining undiagnosed. Type 2 diabetes (T2DM) represents the most prevalent form, accounting for 90% of all diabetes cases.^1^ Alongside lifestyle factors, another reason that contributes to the increasing prevalence of T2DM is aging, which also contributes to the increased risk of dementia. As a result, the finding of people living with T2DM and dementia is very common.^2^

Given the potentially life-threatening acute and chronic complications associated with diabetes, there has been increasing emphasis on improving diabetes management strategies. Many pharmacological interventions are now available to better manage diabetes and its complications, ranging from insulin therapies^3^ to more recent glucagon-like peptide 1 receptor agonists (GLP-1 RAs).^4,5^ However, a challenge that remains is how to better monitor glucose levels in people with diabetes. In this respect, the advent of continuous glucose monitoring (CGM) sensors has been groundbreaking.

### Continuous Glucose Monitoring—Background

A continuous glucose monitoring sensor is a medical device that tracks interstitial glucose levels in real-time. Unlike traditional fingerstick glucose meters that provide single-point readings, CGM systems provide continuous measurement of glucose. Continuous glucose monitoring systems consist of three main parts: a sensor including a microneedle, a transmitter with Bluetooth, and a separate receiver to display the readings. Today, the two main types of CGM systems are: real-time CGM (rtCGM) and intermittent CGM (isCGM). In this article, we will only focus on rtCGM systems. Real-time CGM systems, such as Dexcom and Medtronic, work by automatically measuring interstitial glucose every 5 minutes throughout the day and night. Table 1 lists the main CGM systems that are commercially available. As can be seen in Table 1, Eversense 365 offers the longest wear time of a sensor, while it has the longest warm-up time, with 24 hours. This sensor model, different from other systems, requires charging the transmitter independently.^6^ The mean absolute relative difference (MARD) is the lowest in Libre 3, which is defined as a value for the accuracy of CGM systems for several uses. In addition to MARD, Libre 3 provides real-time glucose measurements every 1 minute, which is different from other systems.^7,8^ For research use, Dexcom G6 and G7 seem to be the only options to have the receiver blinded while also having the shortest warm-up time of 30 minutes.^9^

**Table 1.** Main Available CGM Systems.^6,7,9,10^

	Dexcom G7	Abbott FreeStyle Libre 3 plus	Simplera Sync-Medtronic	Eversense 365
**Maximum wear duration**	10 days	15 days	6 days	365 days
**Warm-up time**	30 min	1 hour	2 hours	24 hours
**FDA-approved sites**	Upper arm, Upper buttocks (for 2-6 years)	Upper arm	Upper arm	Upper arm
**Approved age range**	≥2 years	≥2 years	≥7 years	≥18 years
**FDA approved for dosing without confirmatory fingerpricks**	Yes	Yes	Not stated	Yes
**Drug interactions**	Hydroxyurea, acetaminophen	Not stated	Hydroxyurea, acetaminophen	Tetracycline antibiotics, sorbitol, mannitol
**Display device**	Smartphone or receiver or insulin pump	Smartphone or reader	Smartphone or insulin pump	Smartphone
**Blindness available**	Yes	No	No	No
**MARD**	8.2%	7.8%	Not stated	8.5%

Continuous glucose monitoring technology has become particularly significant in diabetes management, in particular for people with type 1 diabetes (T1DM). Approximately 30% of people with T1DM are using CGM regularly^11^ and it has been shown to be highly beneficial for their diabetes management. However, the same is not the case for T2DM, where CGM uptake has been much slower, for various reasons such as high costs, social anxiety due to the visibility of sensor and technological limitations.^12^ This is surprising since the number of people with T2DM is much higher and often includes older, vulnerable people, who often require often closer monitoring of glucose levels to avoid adverse events. This is particularly true for older people with cognitive changes or comorbid dementia, since these conditions often preclude them from self-monitoring their glucose levels appropriately using capillary blood glucose (CBG) testing, leading to hyper- and hypoglycemic events and further health complications. The focus of our review is T2DM specifically.

### CGM Use in T2DM—Community Settings

The limited research on CGM use in T2DM has mainly been conducted in the community. For example, Ahn et al^13^ conducted a mixed-methods approach involving 30 participants, with an average age of approximately 75 years. Participants wore two CGM devices, and their experiences, along with survey data, were analyzed to gauge CGM usage in managing diabetes in this older T2DM population. The study demonstrated that CGM is highly usable and acceptable among older individuals with diabetes. Participants demonstrated an 81% adherence rate to CGM, indicating effective self-management and decision-making capabilities in diabetes treatment. Participants effectively used CGM to monitor and predict blood glucose trends, positively influencing their glucose control and lifestyle. Satisfaction with CGM averaged 3.76 out of a maximum score of 5, reflecting a balanced perspective of its advantages and inconveniences, such as cost and accuracy concerns. In this regard, the study particularly highlighted the importance of developing CGM educational programs specifically designed for older people, to enhance understanding and effective use. Similarly, they also recommended educating health care professionals in CGM usage and expanding insurance coverage for CGM in non-state-funded health systems.

To our knowledge, there is only one study that investigated CGM use in the community in older people who also had cognitive impairment or dementia. Mattishent et al^14^ conducted a mixed-methods feasibility study in community dwellings in the United Kingdom, involving 12 people aged ≥65 with diabetes and abbreviated mental test score ≤8 or known dementia, using FreeStyle Libre (FSL) CGM. A flash CGM data capture across 14 days ranged between 3 and 92% (mean 55%); 6 participants had <60% capture. This was much lower than in Ahn et al, illustrating the real-world difficulties that older people with memory problems and their caregivers face in glucose self-monitoring, with significant variation in how effectively participants could use the technology. However, the findings also highlighted that the technology was safe to use in this vulnerable population and supported the potential value of CGM for detecting hypoglycemia in those at high risk. In summary, they showed that while participants found wearing the sensor acceptable, data capture varied depending on how many times the reader was used to scan the sensor during the study period, highlighting the need for enhanced support systems and potentially different approaches to CGM implementation in older adults with memory problems.

In terms of hypoglycemic events, a few studies have investigated whether CGM use at home, in care homes, or in long-term facilities can reduce the number of hypoglycemic episodes, which often lead to the highly prevalent falls in older people.^15,16^ A prospective randomized clinical trial was conducted to evaluate the efficacy of rtCGM in adjusting therapy in long-term facilities.^16^ The participants (n = 100) wore blinded CGM for a minimum of 10 days and up to 60 days, with treatment adjusted on CBG point-of-care testing (POCT) in long-term care facilities such as in nursing home care or rehabilitation. The results suggest that the use of rtCGM is safe and effective in guiding insulin therapy in patients with T2DM and captures more readings out of target compared with POCT.^16^ In addition, an rtCGM initiation was associated with a ~3.4% reduction in hypoglycemic events in people living with type 1 and type 2 diabetes.^17^

There is also strong evidence showing that CGM use reduced hospital admissions due to diabetes emergencies.^18-20^ The RELIEF study^18^ is a retrospective study using the French national claims database. A total of 74.011 participants on self-monitoring of blood glucose and prior to starting FSL were recorded for the 12 months before and after initiation. Results showed that hospitalization due to diabetes complications, diabetic ketoacidosis (DKA), and diabetes-associated comas decreased in T1DM and T2DM with the use of FSL at follow-up. Hospital admissions due to hypoglycemia declined by −10.8% in T2DM. Deshmukh et al^19^ conducted a real-world CGM experience study on the impact on glycemic control, hypoglycemia, and hospitalization. In this research, 10.370 (97% with T1DM) FSL user data with follow-up were collected via 102 National Health Service (NHS) hospitals in the United Kingdom. FSL users had a −5.2 mmol/mol change in HbA1c after 7.5 months. Importantly, the hypoglycemic unawareness score declined from 2.7 to 2.4 at follow-up (*P* < .0001). Furthermore, the use of FSL significantly reduced diabetes distress, hospital readmissions, and paramedic calls due to diabetes emergencies.

While glucose-lowering medications and poor-controlled diabetes are linked to increased number of hypoglycemic events and associated hospital admissions due to diabetes emergencies and related secondary complications such as falls, fractures and altered consciousness,^21-23^ CGM use has been shown to lead to less acute complications and a decreased number of hospitalizations, along with an improved time-in-range (TIR) and reduced glycemic variability (GV).^21,22^ The aim for CGM-derived time in range (TIR: 70-180 mL/dL) for older people or those at high risk for any reason is >50%, while the general population with diabetes is recommended to have >70% at target^5,24^ (Table 2). However, there are settings where CGM has been scarcely used despite its potential benefits. One such setting is among hospitalized patients with type 2 diabetes.

**Table 2.** Consensus on Time in Range.^24^

	Time in range 70-180 mg/dL	Time below range <70 mg/dL	Time below range <54-70 mg/dL	Time above range >180-250 mg/dL
Older/high-risk T1DM/ T2DM^a^	>50%	<1%	~0%	<50%
General T1DM/T2DM	>70%	<4%	<1%	<25%

a Older and/or high-risk people with T1DM/T2DM are classified at higher risk for severe hypoglycemia due to age, duration of diabetes, duration of insulin therapy, greater prevalence of hypoglycemia unawareness as well as those with cognitive and physical impairments and other comorbidities and those requiring assisted care.24

Since diabetes-related reasons have an important impact on causes for hospitalization in older people with T2DM (18.8% diabetes-related chronic complications and 0.3% hypoglycemia),^25^ there is an increasing interest as to whether CGM can potentially avoid such dysglycemia and associated hospitalization. A 12-week feasibility study protocol anticipates that CGM will potentially be acceptable to care home residents and at the same time will de-intensify their medication based on the strong link between increased hospitalization and overprescribing.^26^ This study aims to fill the gap in the literature on the evaluation of glucose ranges in the de-intensification process among older individuals. Importantly, the study aims to involve care home residents with or without mental capacity, to further investigate whether in such highly vulnerable older adults can get benefit from CGM. While the previous community evidence shows that CGM is feasible in older people and even those with dementia,^14^ it remains to be shown in this upcoming study whether CGM is also feasible and acceptable to older people in care home setting and whether this might affect their medication de-intensification.^26^ Similar to the previous studies, this study will investigate if there is adequate CGM training in health and care home providers. Continuous glucose monitoring educational programs tailored for older people and health care professionals have been recommended in previous studies,^13,27,28^ highlighting that effective training in CGM is urgently needed in community settings.

### CGM Use in T2DM—Hospital Settings

Only a few randomized controlled studies, especially in older inpatients, have assessed CGM in-hospital settings, and there have been several studies that examine the use of CGM in-hospital. However, despite the low uptake, evidence indicates benefits for glucose control and reduced length of stay among the older population.^29,30^ For example, a multicenter randomized controlled trial comparing sensor-augmented pump (SAP) technology (Medtronic MiniMed Paradigm 722 system) versus multiple daily injection (MDI) with CGM (Medtronic MiniMed CGM System Gold) for a 2-week period in hospitalized older adults with T2DM showed that SAP versus MDI therapy significantly reduced the time required to achieve glycemic targets compared with standard practice. Despite CGM not being the main focus of the study, it highlighted that it can be used in this setting to achieve better glycemic control in hospitalized older people with T2DM. The faster time to target glucose levels suggests potential benefits for reducing the length of hospital stay and improving overall diabetes management in the acute care setting. As an important note, it is essential to consider existing guidelines and position statements from leading professional organizations, including the American Diabetes Association (ADA), Endocrine Society, American Association of Clinical Endocrinology (AACE), and JBDS-IP.^28,31^

To our knowledge, there are only a few randomized controlled trials in the hospital setting that have been conducted. One is DIATEC, which recruited 166 non-intensive care unit (ICU) inpatients with T2DM. The study compared the glycemic outcomes from CGM (Dexcom G6) and POC glucose testing. All participants received CGM; however, POC-arm participants (n = 82) had a blinded CGM for outcome analysis. The results showed no heterogeneity of treatment effect, recommending that all hospitalized individuals had more benefit from CGM compared with POC glucose testing in terms of glycemic outcomes including TIR, time above range, time below range, hypoglycemic events, and mean glucose value.^32^ Similarly, another randomized controlled trial in a non-ICU hospital setting (n = 110) among people living with T2DM has been conducted. According to the results, rtCGM reduced mean glucose by approximately 18.5 mg/dL, increased TIR (70-250 mg/dL) by 11.3%, and was associated with very low and non-increased hypoglycemia in a non-ICU hospital setting.^20^

Based on these existing findings, there are current guidelines for hypoglycemia in-hospital settings such as the ADA and the Joint British Societies for Inpatient Care (JBDS-IP)-UK.^33,34^ The target glucose in the hospital setting is recommended to be between 6 and 12 mmol/L for older and frail inpatients.^31^ It is also suggested to use CGM without alarms at the target for predictive alerts in the hospital wards due to the burden of high frequency of alarms^28^ and possible confusion for patients and the clinical care team.^4,17^ By contrast, usage of CGM for hyperglycemic emergencies is not recommended due to lack of evidence in the accuracy of CGM in rapid changes in glucose and on a fixed or variable rate intravenous insulin infusion.^28^ In this case, instead of CGM, a CBG check is suggested in the hospital.^28^ For example, during DKA, the average glucose was slightly lower than capillary glucose via point-of-care tests among 10 participants.^35^ More trials are needed to improve the accuracy of CGM during hyperglycemic emergencies.

### Conclusion, Implications, and Future Directions

According to community setting studies, CGM is highly feasible and acceptable for older people with diabetes^13^ with a good adherence rate to CGM.^13,14^ The only study in the community dwellings in the United Kingdom with people having diabetes and cognitive impairment showed that the use of CGM is safe and acceptable in this frail group, with potential benefits for capturing asymptomatic hypoglycemia.14 Despite growing interest, CGM usage within hospital settings is still relatively low, despite preliminary evidence that CGM can reduce hospital stays and potential hospital outcomes. These findings suggest that CGM usage in older people, and particularly those with cognitive deficits, might have significant benefits that need to be further explored.

This is particularly relevant for people with dementia, because it is well-established that people living with dementia have a longer length of hospital stay and they occupy 1 in 4 hospital beds.^36^ The most common reasons for hospital admission in this population include falls and acute illnesses. As people get older, they are more likely to fall with ~30% of individuals above 65 years likely to fall and this rises to about 50% for those above 80.^37^ While some falls are likely caused by physical health change, such as muscle weakness, osteoporosis, lower body weight, body mass index (kg/m^2^) <19, poor balance, and visual impairments; other falls are likely caused by hypoglycemia, particularly among individuals with diabetes.^37^ Therefore, considering the increased fall risk among older people with diabetes and the association between diabetes and dementia, this population requires closer glycemic monitoring, for which CGM would be ideal. Usage of CGM in people with dementia and diabetes might decrease the hospitalization rates, potentially help to improve glycemia, and shorten the length of stay.

To our knowledge, there is still an evidence gap in terms of CGM trials and feasibility studies for in-hospital settings among older people living with diabetes, and particularly those with cognitive impairment and/or dementia. Therefore, future studies should develop more evidence for in-hospital settings, while expanding the community-based research. An encouraging approach is to expand the use of CGM in-hospital settings, particularly among older people with diabetes, regardless of type, and those at high risk for dementia. In this vulnerable population, where inpatient care is especially significant, it is important to weigh both the benefits and the challenges of CGM.^38^ Success in this vulnerable population will depend on individual needs, involvement of health care professionals and carers, as well as access to tailored CGM training. Based on this, a personalized approach should be considered to refine best practices in this population.

On the other side, there is limited evidence on how CGM data are managed in older adults with cognitive impairment, including caregiver roles and their impact on treatment decisions. Further research is needed to address some critical aspects of data management and clinical decision-making to ensure that the practical use of CGM will be safe in this population.

In conclusion, our brief review indicates that the use of CGM in older populations remains underdeveloped, despite its considerable potential health benefits. There is emerging evidence showing that CGM is feasible in older people, including those with dementia or mild cognitive impairment in the community. Using CGM in those population can clearly improve glucose levels and reduce hypoglycemic falls and hospital admissions.^18-23^ There is, therefore, clear evidence that CGM use in the community should be encouraged, if appropriate training is available for users.
